# Exogenous γ-aminobutyric acid (GABA) mitigated salinity-induced impairments in mungbean plants by regulating their nitrogen metabolism and antioxidant potential

**DOI:** 10.3389/fpls.2022.1081188

**Published:** 2023-01-18

**Authors:** Abd Ullah, Iftikhar Ali, Javaria Noor, Fanjiang Zeng, Sami Bawazeer, Sayed M Eldin, Muhammad Ahsan Asghar, Hafiz Hassan Javed, Khansa Saleem, Sami Ullah, Haider Ali

**Affiliations:** ^1^ Xinjiang Key Laboratory of Desert Plant Root Ecology and Vegetation Restoration, Xinjiang Institute of Ecology and Geography, Chinese Academy of Sciences, Urumqi, China; ^2^ Cele National Station of Observation and Research for Desert-Grassland Ecosystems, Cele, China; ^3^ University of Chinese Academy of Sciences, Beijing, China; ^4^ Center for Plant Sciences and Biodiversity, University of Swat, Charbagh Swat, Pakistan; ^5^ Department of Genetics and Development, Columbia University Irving Medical Center, New York, NY, United States; ^6^ Department of Botany, Islamia College University, Peshawar, Pakistan; ^7^ Umm Al-Qura University, Faculty of Pharmacy, Department of Pharmacognosy, Makkah, Saudi Arabia; ^8^ Center of Research, Faculty of Engineering, Future University in Egypt, New Cairo, Egypt; ^9^ Department of Biological Resources, Agricultural Institute, Centre for Agricultural Research, ELKH, 2 Brunszvik St. Martonvásár, Hungary; ^10^ College of Agronomy, Sichuan Agricultural University, Chengdu, China; ^11^ Department of Horticultural Sciences, The Islamia University of Bahawalpur, Bahawalpur, Pakistan; ^12^ Department of Botany, University of Peshawar, Peshawar, Pakistan

**Keywords:** Salinity, plant abiotic stress, plant growth regulators, phytohormones, physiologicalmechanism, antioxidant mechanism, nitrogen metabolism

## Abstract

**Background:**

Increasing soil salinization has a detrimental effect on agricultural productivity.Therefore, strategies are needed to induce salinity-tolerance in crop species for sustainable foodproduction. γ-aminobutyric acid (GABA) plays a key role in regulating plant salinity stresstolerance. However, it remains largely unknown how mungbean plants (Vigna radiata L.) respondto exogenous GABA under salinity stress.

**Methods:**

Thus, we evaluated the effect of exogenous GABA (1.5 mM) on the growth and physiobiochemicalresponse mechanism of mungbean plants to saline stress (0-, 50-, and 100 mM [NaCland Na2SO4, at a 1:1 molar ratio]).

**Results:**

Increased saline stress adversely affected mungbean plants' growth and metabolism. Forinstance, leaf-stem-root biomass (34- and 56%, 31- and 53%, and 27- and 56% under 50- and 100mM, respectively]) and chlorophyll concentrations declined. The carotenoid level increased (10%)at 50 mM and remained unaffected at 100 mM. Hydrogen peroxide (H2O2), malondialdehyde(MDA), osmolytes (soluble sugars, soluble proteins, proline), total phenolic content, andenzymatic activities of superoxide dismutase (SOD), ascorbate peroxidase (APX), peroxidase(POD), glutathione reductase (GTR), and polyphenol oxidation (PPO) were significantlyincreased. In leaves, salinity caused a significant increase in Na+ concentration but a decrease inK+ concentration, resulting in a low K+/Na+ concentration (51- and 71% under 50- and 100- mMstress). Additionally, nitrogen concentration and the activities of nitrate reductase (NR) andglutamine synthetase (GS) decreased significantly. The reduction in glutamate synthase (GOGAT)activity was only significant (65%) at 100 mM stress. Exogenous GABA decreased Na+, H2O2,and MDA concentrations but enhanced photosynthetic pigments, K+ and K+/Na+ ratio, Nmetabolism, osmolytes, and enzymatic antioxidant activities, thus reducing salinity-associatedstress damages, resulting in improved growth and biomass.

**Conclusion:**

Exogenous GABA may have improved the salinity tolerance of mungbean plants by maintaining their morpho-physiological responses and reducing the accumulation of harmfulsubstances under salinity. Future molecular studies can contribute to a better understanding of themolecular mechanisms by which GABA regulates mungbean salinity tolerance.

## 1 Introduction

Soil salinity is a major abiotic factor that impedes agricultural productivity. Around 3600 million hectares (Mha) of arable land are degraded due to salinization, causing a loss of approximately USD 27.5 billion annually (Zhang and Shi, 2013; Qadir et al., 2014). As a consequence of global warming, environmental fluctuations, industrial pollution, unsustainable use of fertilizers, and irrigation using salt water, the problem of salinization is expected to worsen in the coming years (Zhu et al., 2019). The demand for food production will increase by 70% as the world population approaches 10 billion by 2050. This will put further pressure on the declining area of arable lands (Calone et al., 2021). Therefore, rapid salinization negatively impacts socioeconomic and ecological development (Sehrawat et al., 2018). Salt-affected soil results in the accumulation of toxic levels of salt ions in plants, for example Na^+^ and Cl^−^ ions, resulting in osmotic stress, ionic toxicity, and nutrient deficiency (Munns and Tester, 2008; Polash et al., 2019; Rahman et al., 2019). Moreover, salinity hinders seed germination, seedling growth establishment (Ullah et al., 2022a; Saeed et al., 2022), nitrogen assimilation and anti-oxidant mechanism and several other key physiological processes (Ma et al., 2018; Ullah et al., 2022b).

The damages of salinity stress can be avoided by plants using several mechanisms, such as (i) the removal of toxic salt ions or their compartmentation within vacuoles or old tissues, (ii) the synthesis of compatible solutes, and (iii) the upregulation of non-enzymatic and enzymatic antioxidant defense mechanisms against salt-induced oxidative stress (Kaya et al., 2010a; Rahman et al., 2019; Mostofa et al., 2021). Salinity stress causes photoinhibition, which leads to an accumulation of untapped energy that destroys the photosynthetic apparatus, resulting in the formation of reactive oxygen species (ROS; Gururani et al., 2015; Polash et al., 2019; Ullah et al., 2022c). Increasing ROS levels weaken the antioxidant apparatus in cells, causing lipid peroxidation (MDA), which affects the membrane permeability and structure of the cell membrane, and causes damage to lipids, proteins, and nucleic acids (Tanveer, 2020). However, plants can upregulate their antioxidant mechanisms, including antioxidant enzymes [i.e. superoxide dismutase (SOD), ascorbate peroxidase (APX), peroxidase (POD), glutathione reductase (GTR), and polyphenol oxidation (PPO), monodehydroascorbate reductase (MDHAR), and dehydroascorbate reductase (DHAR)] and metabolites [i.e. ascorbate (AsA) and glutathione

(GSH)] to reverse hydrogen peroxide (H_2_O_2_) (Kang et al., 2013; Kaya et al., 2020a). The anti-oxidant mechanism is, therefore, positively correlated with the salt tolerance of plants. Furthermore, plants use a greater fraction of their carbon and energy in stress-coping mechanisms, such as forming compatible solutes to maintain salinity homeostasis (Slama et al., 2015; Sami et al., 2016; Asrar et al., 2017).

These osmolytes prevent water loss and chlorophyll degradation, regulate cell division and expansion, stabilize proteins, eliminate excess ROS, maintain osmotic balance, prevent ionic toxicity, and regulate certain genes (Verdoy et al., 2006; Sami et al., 2016; Ahmad et al., 2016; Ullah et al., 2022b). Nitrogen (N) is an essential component of photosynthetic capacity, which enables plants to grow and develop optimally. Salinity impacts N metabolism by inhibiting its metabolizing enzymes such as nitrate reductase (NR), glutamine synthetase (GS), and glutamate synthetase (GOGAT) enzymes (Debouba et al., 2006; Meng et al., 2016; Ullah et al., 2019; Ullah et al., 2022b). It is believed that N metabolism plays a profound role in the ability of plants to withstand salt stress. Nevertheless, the relationship between N metabolism and salinity is complex. It depends on several factors, including the level and duration of salt stress, the availability, type and source of N in the soil (Munns and Tester, 2008; Dai et al., 2015).

Mung bean (*Vigna radiata* L.) is a short-lived leguminous economic legume crop, rich in carbohydrates, protein, fibres, vitamins, fatty acids, minerals, and essential amino acids (Sehrawat et al., 2018). Approximately 3 million tons of mung beans each year, which accounts for 5% of the total production of pulses worldwide (World Vegetable Centre, 2018). Nevertheless, legumes such as mung beans are typically grown in arid and semi-arid regions where salt is a problem, thus making them salt-sensitive species (Sprent and Gehlot, 2010). Previous studies on mungbean plants reported that salinity affects their germination and seedling growth, plant growth and biomass, photosynthesis, nutrient acquisition, relative water content (RWC), ROS production, membrane stability, photosynthetic chlorophyll, and carotenoid pigments, root hair formation, nodule respiration and nodulation (review; Sehrawat et al., 2018). Moreover, its yield has been reported to drop to almost 70% under 50 mM NaCl, negatively affecting crop quality (Sehrawat et al., 2018). Considering the low productivity of mungbean, it is necessary to improve its salt tolerance to maintain its production in salt-affected soils. Therefore, it is imperative to examine its growth and adaptive physio-biochemical mechanism in response to salinity stress.

γ-Aminobutyric acid (GABA) is a nonprotein amino acid containing four highly water-soluble carbons. Mitochondria synthesizes GABA *via* the GABA shunt. Plants rapidly accumulate GABA in response to several abiotic stress factors (Mekonnen et al., 2016; Shelp et al., 2017; Sita and Kumar, 2020). Various plant growth regulators, including GABA, have been found to provide a level of tolerance to salinity through the modulation of physiological responses to the unfavorable environment (Ma et al., 2018; Jin et al., 2019; Khanna et al., 2021). Exogenous GABA has been demonstrated to enhance plant adaptation to abiotic stress conditions through improvements in growth, photosynthesis, enzymatic and non-enzymatic antioxidative defense mechanisms, and nitrogen metabolism (Beuve et al., 2004; Vijayakumari et al., 2016; Ma et al., 2018; Salah et al., 2019). Even though various studies have been conducted on the effects of salinity stress on mung beans, the effect of GABA application has largely been overlooked. For instance, a recent study on mung beans regarding GABA application under salinity focused only on its role in seed germination (Ji et al., 2020). Accordingly, GABA research needs to intensify the focus on legumes in general and mung beans in particular. We hypothesized that saline stress would hinder the metabolism of mungbean plants, but exogenous GABA may improve their morpho-physio-biochemical damages by reducing the adverse effects of saline stress. To test our hypothesis, we examined the effects of exogenous GABA application (1.5 mM) on growth, photosynthetic pigments, osmolytes, minerals regulation, nitrogen metabolism, lipid peroxidation, and antioxidant enzymes in saline-stressed (0-, 50-, and 100 mM) mung bean seedlings.

## 2 Materials and methods

### 2.1 Experiment design

The experiment was conducted in a greenhouse environment (controlled condition) at the Department of Botany, University of Peshawar, Peshawar, 25120 (34◦15 North latitude and 71◦42 East longitudes), KP Pakistan. Temperatures typically range between 5°C and 39°C from January to February and June to July, respectively, with an average rainfall of approximately 513 mm per year. The silt-loamy soil had pH 6.9, a bulk density of 1.55 g cm^−3^, and EC 0.288 ds/m, collected from the experimental site. The seeds of local mungbean accession (*Vigna radiata* L.) were provided by Cereal Crop Research Institute (CCRI), Persabaq, Nowshera 24050, Pakistan. These seeds were sown in pots (15 cm in diameter) with an opening at the bottom (2 cm diameter) and filled with 2.5 kg of silt-loam soil.

### 2.2 Salinity treatments and γ-Aminobutyric acid (GABA) application

In the beginning, the seedlings were watered every three hours with tap water. At the first trifoliate leaf stage (three weeks after sowing), 48 pots (2 seedlings per pot) with uniform seedlings were selected and divided into six sets for the application of saline stress (SS; NaCl and Na_2_SO_4_, 1:1 molar ratio) and GABA application. There were three saline stress treatments: 0-mM (control), 50-mM, and 100-mM. The remaining three groups were subjected to the same levels of saline stress but were given exogenous GABA solutions (1.5 mM, 200 ml; 100 ml GABA applied to each pot at 20- and 30-days of sowing, respectively). An initial seedlings growth experiment was conducted with GABA concentrations ranging between 0.25mM and 1.5mM (**Supplementary Table 1**). The optimal GABA concentration was selected based on the improved growth of mungbean seedlings subjected to 50 mM saline stress. Three replicates of each treatment were conducted. We harvested the 45-day-old mung bean plants and immediately froze them in liquid nitrogen before storing them at -80°C for physiologic analysis.

### 2.3 Measurement of plant growth and biomass

The stem height and root length were measured using a measuring tape. Next fresh and dry weights of the stem, leaves, and root of the seedlings were measured using an electric balance. For dry weight determination, the plants were oven-dried at 105°C for 30 min and then dried at 75°C until constant weight.

### 2.4 Measurement of photosynthetic pigments

We extracted photosynthetic chlorophyll pigments (0.1–0.3 g) from the fresh leaf samples (0.1–0.3 g) using ethanol (95%, vol/vol) following a standard method (Lichtenthaler and Buschmann, 2001). The absorbances were read at 665 nm and 649 nm using a spectrophotometer. Chlorophyll concentrations were calculated using the following equations (mg g^−1^ FW).


(1) Chl a = 13.98 A665 – 6.88 A649 

(2) Chl b = 24.96 A649 – 7.32 A665

(3) Chl a/b = Chl a / Chl b

(4) Chl = Chl a + Chl b 

### 2.5 Determination of mineral elements

We digested the leaf sample (0.05 g) with concentrated HNO_3_ (3 mL). Afterwards, the extract was brought up and diluted to 15 mL with deionized water. An inductively coupled plasma-optical emission spectrometry (ICP-OES) was used to determine Na^+^ and K^+^ concentrations, following a standard method (Mostofa et al., 2015).

### 2.6 Determination of H_2_O_2_ and MDA concentration

A standard procedure (Patterson et al., 1984) was used to determine hydrogen peroxide (H_2_O_2_). Using a chilled mortar, fresh leaf samples (0.2 g) were homogenized in 5 ml of trichloroacetic acid (TCA) (0.1%) in an ice bath. In the following step, the extract was centrifuged at 5000 × g for 10 min (4°C). Next, the supernatant containing the titanium reagent (50 ml of 20% titanium tetrachloride in ammonia) and titanium reagent was centrifuged at 10,000 × g for 10 min. After five washes with acetone, the precipitate was centrifuged at 10,000 g for 10 min, followed by adding 3 ml of 1 M H_2_SO_4_.

For the evaluation of lipid peroxidation, malondialdehyde (MDA) concentrations were determined using the thiobarbituric acid (TBA) test (Heath and Packer, 1965). We homogenized fresh leaf samples (0.5 g) in 1ml of 5% TCA and centrifuged them for 10 min at 5,000 × g (4°C). We then added 4 ml of the supernatant to two ml of 20% TCA in a separate tube and heated this mixture at 100°C for 15 min before centrifuging it at 5,000 g (10 min). The absorbance at 450, 532, and 600 nm were read using a spectrophotometer, and MDA concentration was determined using the equation below.


$$MDA\;\left(mol\;g^{-1}FW\right)\;=\;=\;6.45\;\left(A532-\;A600\right)\;–\;0.56A450.$$

### 2.7 Antioxidant Enzyme Activities

A chilled mortar was used to grind and homogenize fresh leaf samples in a 0.1 M phosphate buffer (pH 7.3) solution and 0.5 mM ethylenediaminetetraacetic acid (EDTA). The Homogenates were centrifuged at 12000 ×g for 10 min (at 4°C). Following this, the supernatant containing enzyme extract was used for the assays. We determined the superoxide dismutase (SOD) activity using a standard method (Giannopolitis and Ries, 1977). Approximately 0.1 mL of enzyme extract was added to a reaction mixture of 50 mM phosphate buffer (pH 7.8), 130 mM of methionine, 2.0 mM of riboflavin, and 75 mM of nitro-blue tetrazolium (NBT). The activity of SOD was determined by measuring the decline rate of nitroblue tetrazolium at a 560 nm wavelength using a spectrophotometer. During the measurement of SOD activity, one unit corresponds to the amount of enzyme required to inhibit 50% of NBT reduction at 560 nm.

Furthermore, POD activity was determined using standard methods (Wang et al., 2018) but with minor changes. An enzyme extract of 0.5 ml was mixed with 2 ml of buffer substrate (guaiacol and Na_3_PO_4_ pH 6.4), 24 mM H_2_O_2_, and 1 ml of buffer substrate. Measurement of absorbance at 460 nm was conducted twice at intervals of 1 min. Enzyme activity was calculated by increasing the absorbance of the reaction system by 0.01 up to a maximum of 1U per min, which was then converted to U/g·min^−1^. The monitoring of H_2_O_2_ disappearance was used to determine CAT activity (Sabra et al., 2012). As a starting point, 50 ml of enzyme extract was dissolved in 1.5 ml of reaction mixture containing 50 mM K-phosphate buffer (pH 7.0) and 15 mM hydrogen peroxide. An absorbance measurement was conducted at 240 nm for 1 min using a spectrophotometer. The degradation of one mole of H_2_O_2_ per minute is equivalent to one unit of CAT. We determined the ascorbate peroxidase activity (APX) using a standard method. (Katsumi et al., 1994). Briefly, a 3 mL reaction mixture that contained 50 mM of phosphate buffer (pH 7.0), 1.0 mM of hydrogen peroxide, 0.25 mM of L-ascorbic acid, and 0.1 mL of enzyme extract was prepared. An increase in absorption, at 290 nm, was observed with a spectrophotometer following ascorbate oxidation. glutathione reductase (GR) activity was determined using a previous method (Carlberg and Mannervik, 1985). An enzyme extract was added to a reaction mixture of 50 mM Tris-HCl buffer (pH 7.5), 0.5 mM GSSG, 3 mM MgCl2, and 0.2 mM NADPH. GSSG was added to initiate the reaction. A spectrophotometer was used to measure the absorbance at 340 nm. One unit of activity corresponds to the amount of glutathione reductase capable of catalyzing the oxidation of one mol of NADPH min^−1.^ The Polyphenol peroxidase (PPO) activity was determined using a standard method (Cañal et al., 1988) with some modifications. We prepared a reaction mixture of 2.8 ml of 100 mM NaPi, pH 7.0, 0.1 ml of 25 mM pyrogallol, and 100 ml of enzyme extract. We maintained the mixture at 30°C for 30 min and measured the activity at 420nm after 30 minutes.

### 2.8 Determination of N-metabolizing enzymes

A sulfamate colorimetric method was used to determine nitrate reductase (NR) activity (Ullah et al., 2019). Briefly, fresh leaf samples (0.3 g) were homogenized in 200 mM KNO3, 5 mM EDTA, and 0.15 mM NADH in 100 mM phosphate buffer (pH 7.5), and the reaction mixture was incubated for 1 hour at 30°C. Next, the reaction mixture was centrifuged at 30,000 × g for 20 min. Next, 2 ml of sulfanilamide and N-naphthylamine reagents were added, and absorbance was read at 540 nm. Units of enzyme activity were expressed as μmol g^−1^ protein. We determined the glutamine synthetase (GS) activity using a standard method (Ullah et al., 2019). Fresh leaf samples (0.3 g) were homogenized in 2 ml of 50 mM Tris-HCl buffer (pH 7.8; containing 0.1% TritonX-100, 15% glycerol, 1 mM EDTA, and 14 mM 2-mercaptoethanol), and incubated at 37 ^◦^C for 30 min. Afterwards, the reaction mixture was centrifuged twice at 4°C for 10 min each time. Following this, 1 mL of ferric chloride reagent was added and centrifuged at 5,000°C for 10 min. At 540 nm, the absorbance was measured, and enzyme activity was expressed as μmol g^−1^ protein. The glutamine synthase (GOGAT) activity was determined according to a standard method (Ullah et al., 2019). The Assay mixture contained100 mM K^+^-ketoglutaric acid, 20 mM L-glutamine, 3 mM NADH, and 10 mM KCl, in 25 mM Tris–HCl (pH 7.2). Next, the enzyme extract was added to initiate the reaction. An absorbance measurement at 340 nm continuously monitored the NADH oxidation. The oxidation of 1 μmol of NADH per minute was considered an enzyme unit (μmol g^−1^ protein).

### 2.9 Determination of biochemical parameters

We measured the soluble sugar concentration using the anthrone method (Yemm and Willis, 1954), and the standard was glucose. The concentration of proline was determined using a standard method (107). We homogenized 0.5 grams of fresh leaf samples in 3 percent aqueous sulfosalicyclic acid and then centrifuged at 5,000 × g (10 min). Next, the filtrate (2 ml) was mixed with 2 ml of glacial acetic acid and 2 ml of acid-ninhydrin in a test tube. Afterward, the reaction mixture was boiled at 100°C for 1 hour. Toluene was used to extract the reaction mixture. We aspirated and cooled the chromophore containing toluene. Finally, a spectrometer was used to measure absorbance at 520 nm. The soluble proteins were determined using 0.3 grams of fresh leaf samples (Bradford, 1976), standard bovine serum albumin. We extracted total phenolic content from dried leaf samples (0.3) in 80% methanol using the Folin-Ciocalteu colorimetric analysis (Scalbert et al., 1989). The absorbance was measured using a spectrophotometer at 765 nm. Phenolic content was expressed as mg g^−1^ DW, dry weight.

### 2.10 Analysis

Three replicates of the measurements were conducted. The descriptive statistics and one-way analysis of variance (ANOVA) were conducted using SPSS version 16.0 (Chicago, IL, United States). At a significance level of *p*<0.05, Duncan’s multiple range tests were used to compare means. The figures were created using GraphPad Prism 8. The growth parameters, chlorophyll pigment content, osmolytes, N metabolism, mineral nutrition, H_2_O_2_ and MDA levels, and antioxidant enzyme activity were examined using Pearson correlation analyses (Origin Lab Corporation, Northampton, MA, USA) for further interpretation.

## 3 Results

### 3.1 Changes in growth and biomass

**Figure 1:**
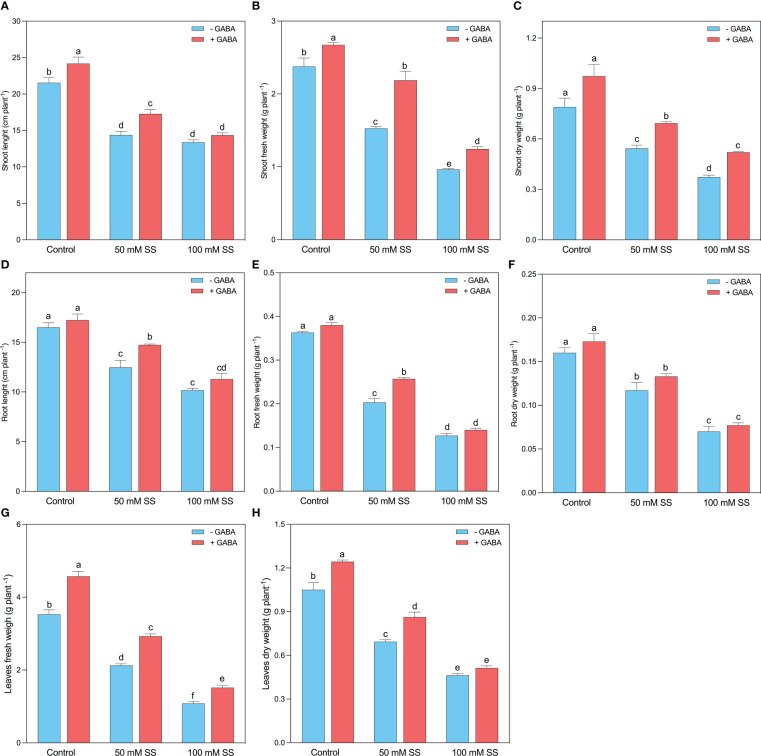
Changes in **(A)** shoot length, **(B)** shoot fresh weight and **(C)** shoot dry weight, **(D)** root length, **(E)** root fresh weight, **(F)** root dry weight, **(G)** leaf fresh weight, and **(H)** leaf dry weight of mungbean plants under saline stress (SS) and exogenous (γ-aminobutyric acid) treatments. The bars indicate the SE of the mean value, n=3. Different letters above the bars indicate significantly different values at *P*<0.05 following Duncan’s method.

Mungbean plants showed obvious differences in growth performance when treated with saline stress and GABA treatments. Both salinity levels (50 mM and 100 mM) substantially reduced the growth parameters of mungbean plants. The shoot length (SL), stem fresh weight (SFW), and stem dry weight (SDW) experienced a 33.3-, 35.8- and 31.2% inhibition following 50 mM SS, while 37.9-, 59.5- and 52.7% decline was found after 100 mM SS, respectively (**Figures 1A–C**). GABA, however, improved the SL, SFW, and SDW under 50 mM (20.2-, 43.2-, and 27.6%, respectively) and 100 mM (27.2-, 29.1-, and 39.3%, respectively) compared to untreated plants. This improvement was 12.2-, 12.5-, and 23.2% when GABA was applied under controlled conditions. Similar to the stem indices, the root length (RL), root fresh weight (RFW), and root dry weight (RDW) declined by 24.4-, 44.0- and 27.1% after 50 mM SS, while following 100 mM SS, this inhibition was 38.4-, 65.1- and 56.3% as compared to the control, respectively (**Figures 1D–F**).

Nonetheless, GABA application significantly improved RL and RFW under 50 mM (18.2- and 26.3%). Further, GABA caused a non-significant increment in RL and RFW under 100 mM and RDW under both stress conditions (**Figures 1D–F**). The leaf morphology was also substantially influenced by saline stress since a 39.9-, 34.0% and 69.3-, and 55.9% inhibition was recorded in leaf fresh weight (LFW) and leaf dry weight (LDW) following the 50 mM and 100 mM SS, respectively. However, GABA significantly improved LFW, and LDW under 0 mM (29.7 and 18.4%, respectively), 50 mM (37.8 and 24.5%, respectively), and 100 mM SS (44.0-, and 10.8%, respectively), compared to untreated plants (**Figures 1G, H**).

### 3.2 Changes in photosynthetic pigments

**Figure 2:**
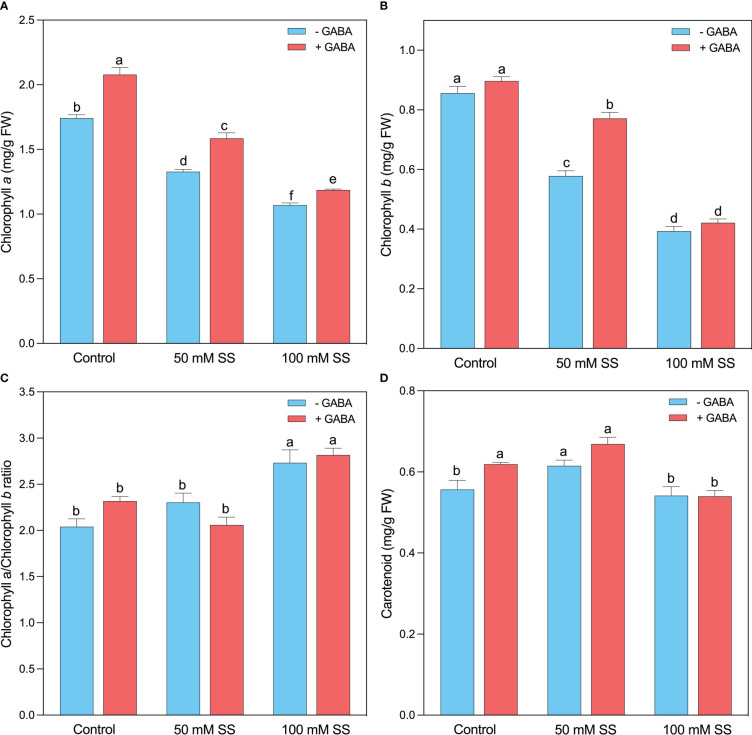
Changes in concentrations of **(A)** chlorophyll a, **(B)** chlorophyll b, **(C)** chlorophyll *a*/*b* ratio **(D)** of mungbean plants under saline stress **(SS)** and exogenous (γ-aminobutyric acid) treatments. The bars indicate the SE of the mean value, n=3. Different letters above the bars indicate significantly different values at *P*<0.05 following Duncan’s method.

The mungbean plants exhibited lower chlorophyll a (Chl a) and chlorophyll b (Chl b) content under salinity stress compared to the un-treated plants since a decline of 24-, 32- and 39- and 54%, following 50 mM and 100 mM, respectively (**Figures 2A, B**). Nonetheless, the supplementation of GABA to both saline levels considerably improved the Chl a and Chl b contents by 19.3-and 5-%; 19- and 33%; and 11- and 7%, respectively. Interestingly, the Chl a/b ratio showed a different behavior, and a significant increase was observed under both salinity levels (**Figure 2C**). A 13- and 34% rise was recorded under 50 Mm and 100 mM SS, respectively. GABA improved (21.6%) the Chl a/b ratio following 100 mM SS (**Figure 2C**). Carotenoid levels significantly increased at 50 mM and remained unaffected at 100 mM saline stress level. Exogenous GABA significantly increased carotenoid level under controlled condition, where it has no profound effect under salt conditions, compared to their untreated peers (**Figure 2D**)

### 3.3 Changes in Na^+^ and K^+^ concentrations

**Figure 3:**
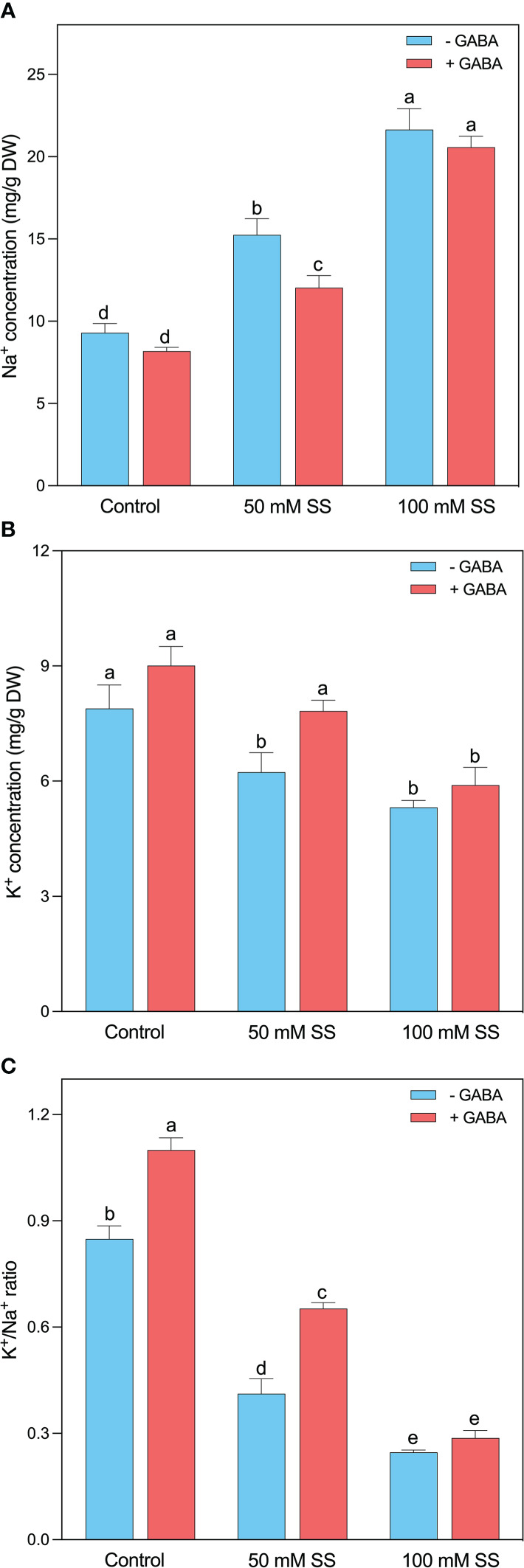
Changes in concentration of leaf **(A)** Na^+^, **(B)** K^+^, and **(C)** K^+/^Na^+^ ratio and root (f) Na^+^, (g) Cl^-^ (h) K^+^ (i) Mg^++^ and (i) K^+/^Na^+^ ratio of mungbean plants under saline stress (SS) and exogenous (γ-aminobutyric acid) treatments. The bars indicate the SE of the mean value, n=3. Different letters above the bars indicate significantly different values at *P*<0.05 following Duncan’s method.

The excess Na^+^ levels strongly indicate saline/alkaline stress persistence in plants. A Similar was the case in this study since a rapid enhancement was noticed after the induction of saline stress at both levels. This increment was 1.6- and 2.3-fold following 50 mM and 100 mM SS, respectively. Although the GABA could not profoundly reduce the Na^+^ levels after 100 mM SS, however, significant inhibition was observed under 50 mM SS (**Figure 3A**).

The exposure to saline stress reduced N content, K^+^ levels, and K^+^/Na^+^ ratio. The mungbean treated with 50 mM SS exhibited 38, 21, and 51% decreases in N content, K^+^ levels, and K^+^/Na^+^ ratio, and this reduction was even greater (59, 33, and 71%) in the case of 100 mM SS, respectively (**Figures 3B, C**). Although, the exogenous application of GABA could help the mungbean plants in the considerable improvement of these parameters under 50 mM SS. Whereas the 100 mM saline-stressed plants did not display such an obvious promotion in these parameters after GABA application (**Figure 3C**).

### 3.4 Changes in H_2_O_2_ and MDA concentration

The H_2_O_2_ and MDA are the major determinants of oxidative stress in plants since the production of these compounds is directly related to the increased cellular damage post oxidative stresses (**Figures 4A, B**). Similarly, in the current experiment, after the induction of oxidative stress caused by saline stress, the H_2_O_2_ and MDA levels were raised by 47 and 86% under 50 mM. This enhancement was 1.9- and 2.1-fold following 100 mM, respectively. Nevertheless, using exogenous GABA declined the H_2_O_2_ and MDA levels by 1.3- and 1.8-fold under 50 mM SS and 1.9- and 2.2-fold after100 mM SS, respectively (**Figures 4A, B**).

**EFigure 4:**
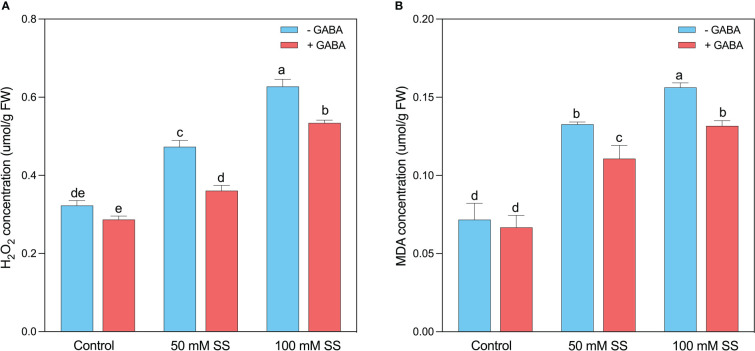
Changes in concentrations of **(A)** H_2_O_2_ and **(B)** MDA of mungbean plants under saline stress (SS) and exogenous (γ-aminobutyric acid) treatments. The bars indicate the SE of the mean value, n=3. Different letters above the bars indicate significantly different values at *P*<0.05 following Duncan’s method.

### 3.5 Changes in the enzymatic antioxidant system

**Figure 5:**
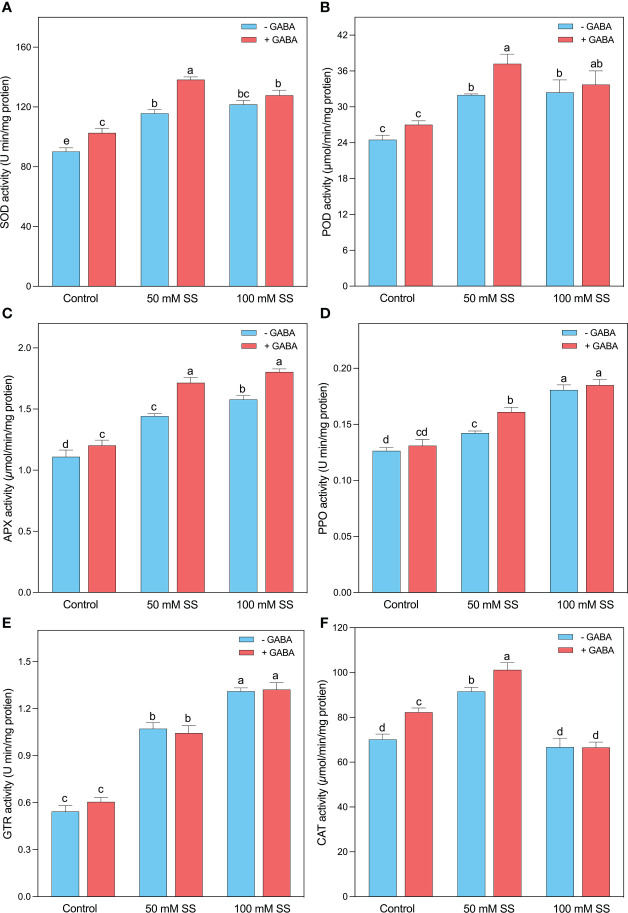
Changes in enzymatic activities of **(A)** SOD, **(B)** POD, **(C)** APX, **(D)** PPO, **(E)** GR, and **(F)** CAT of mungbean plants under saline stress (SS) and exogenous (γ-aminobutyric acid) treatments. The bars indicate the SE of the mean value, n=3. Different letters above the bars indicate significantly different values at *P*<0.05 following Duncan’s method.

Salt-induced higher concentrations of H_2_O_2_ and MDA, the mungbean plants tremendously stimulated their antioxidant potential to scavenge excessive ROS. Briefly, the SOD, POD, APX, PPO, and GTR activities were enhanced by 1.3-, 1.3-, 1.3-, 1.16-, and 2-fold when the plants were grown under 50 mM SS, while the 100 mM SS-treated mungbean plants displayed 1.3-, 1.3-, 1.4, 1.5-, and 2.4-fold stimulation after 100 mM SS, respectively (**Figures 5A–E**). The exogenous application of GABA further improved the antioxidant activities of POD and SOD by 38 and 35% at 50 mM SS levels, while the APX, PPO, and GTR increments were 1.5-, 1.4-, and 2.2-fold after 100 mM SS, respectively (**Figures 5A–E**). Intriguingly, the CAT activity significantly increased under 50 mM (30.4%) compared to the control condition. However, GABA application significantly enhanced under 0 mM and 50 mM (17.3- and 10.5%, respectively) compared to their untreated peers. However, SS and GABA had no significant effect on CAT activity (**Figure 5F**)

### 3.6 Changes in nitrogen metabolizing enzymes

**Figure 6:**
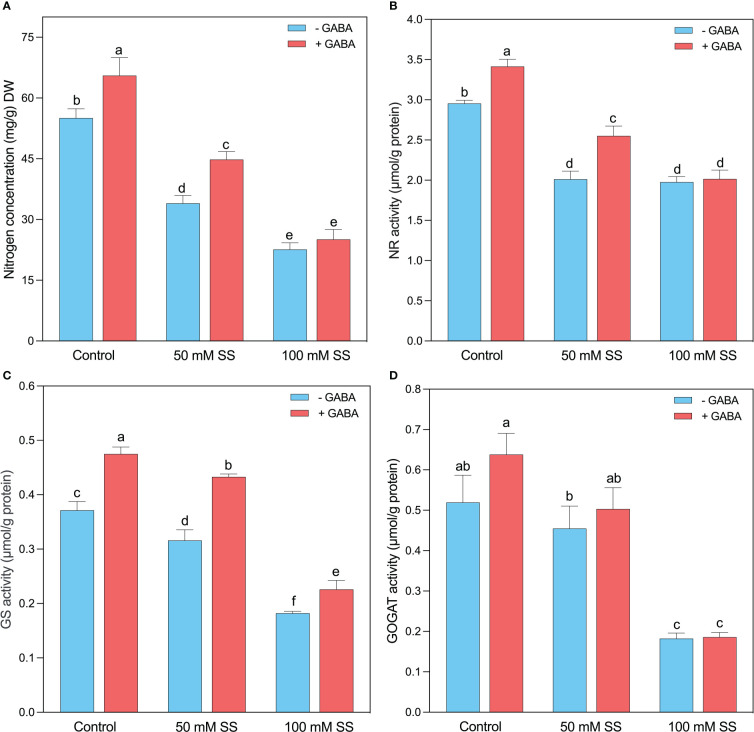
Changes in concentrations of **(A)** N concentration and enzymatic activities of **(B)** NR, **(C)** GS, and **(D)** GOGAT of mungbean plants under saline stress (SS) and exogenous (γ-aminobutyric acid) treatments. The bars indicate the SE of the mean value, n=3. Different letters above the bars indicate significantly different values at *P*<0.05 following Duncan’s method.

The nitrogen (N) concentration significantly decreased under 50 mM and 100 mM by 38.3- and 58.9%, respectively, compared to the control (**Figure 6A**). Compared to untreated peers, exogenous GABA significantly increased N concentration under 0 mM and 50 mM (19 and 31.8%, respectively) but had no significant effect under 100 mM SS. Intriguingly, the NR (32-, 33.1%), GS (15- and 51%), and GOGAT (12.5- and 65%) were significantly reduced by the induction of 50- and 100 mM SS (**Figures 6B–D**). However, GABA significantly improved NR under 0- and 50 mM SS (16- and 27-%, respectively), whereas GS under 0-, 50- and 100 mM SS (28- and 37-, and 24.2%, respectively), compared to controlled conditions. Moreover, GABA had no significant effect on GOGAT under controlled and SS conditions (**Figures 6B–D**).

### 3.7 Changes in biochemical contents

**Figure 7:**
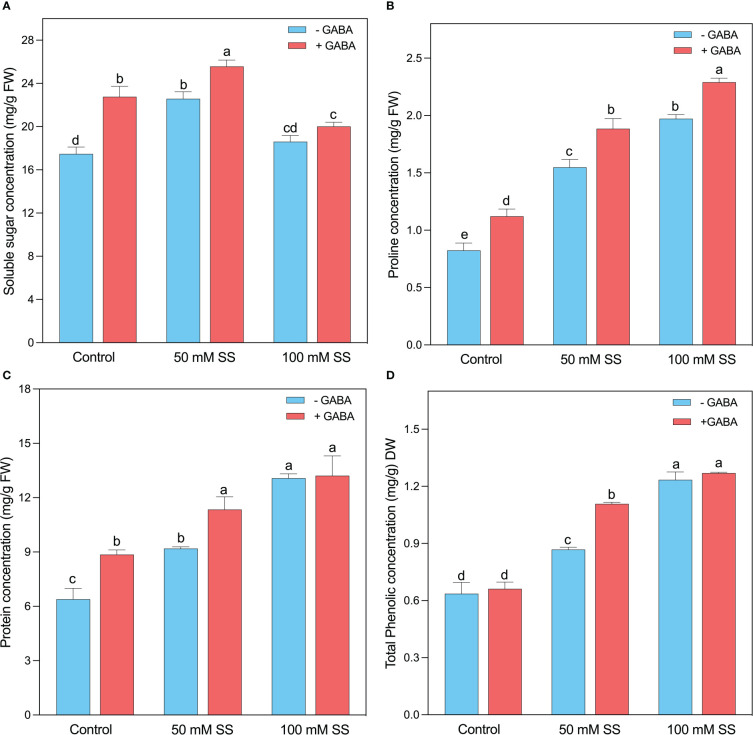
Changes in concentrations of **(A)** sugar, **(B)** soluble protein, **(C)** proline, and **(D)** total phenolic content of mungbean plants under saline stress **(SS)** and exogenous (γ-aminobutyric acid) treatments. The bars indicate the SE of the mean value, n=3. Different letters above the bars indicate significantly different values at *P*<0.05 following Duncan’s method.

The soluble sugar concentration increased under saline stress and GABA application. For instance, it increased by 29.2- and 6.5% under 50- and 100 mM SS (**Figure 7A**). Further, the GABA application enhanced soluble sugar by 30.3-, 13.3-, and 7.6% under 0-, 50-, and 100 mM SS. Interestingly, the protein content significantly improved at both GABA-treated and untreated treatments, irrespective of the salt stress. Generally, 44- and 28% of increment was observed after 50 mM of salinity stress in untreated and treated plants, respectively (**Figure 7B**). Similarly, both GABA-treated and untreated mungbean plants showed a 1.5- and 2-fold increase after 100 mM SS, respectively. Like enzymatic antioxidants, the non-enzymatic antioxidant compounds, such as proline, and phenol, were considerably influenced by the salt stress in mungbean plants (**Figures 7C, D**). When the plants were exposed to 50 mM SS, the proline, and total phenolic content, were enhanced by 88- and 36% and the GABA application further improved them by 68- and 66 4% under 50 mM SS, respectively. In addition, the mungbean plants under 100 mM salinity stress showed a 2.4- and 2- fold rise, and a further 2-and 1.9-fold increase was deployed by the GABA supplementation, respectively (**Figures 7C, D**).

### 3.8 Correlation analysis

**Figure 8:**
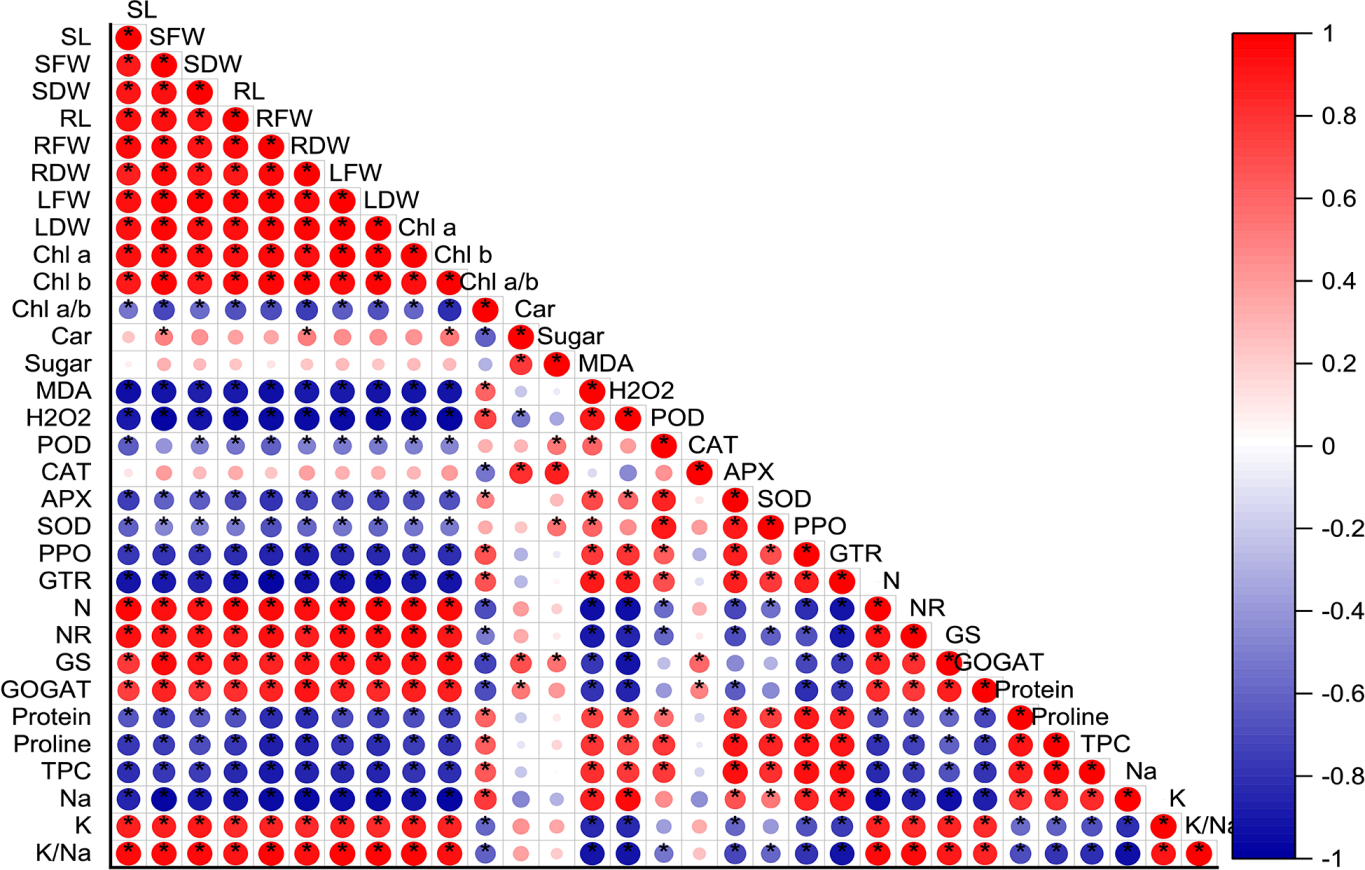
Correlation analysis between all the studied parameters. Red and blue color represent the positive and negative correlation. The size and intensity of color exhibited the significance of variables, and * represents the significance level at *P*<0.05. SL, stem length; SFW, stem fresh weight; SDW, stem dry weight; RL, root length, RFW, root fresh weight; RDW, root dry weight, LFW, Leaf fresh weight; LDW, leaf dry weight; Chl, chlorophyll; Car, carotenoids; MDA, malondialdehyde; H_2_O_2_, hydrogen peroxide; POD, peroxidase; CAT, catalase; APX, ascorbate peroxidase; SOD, superoxide dismutase; PPO, polyphenol oxidases; GTR, glutathione reductase; N, nitrogen; NR, nitrate reductase; GS, glutamine synthetase; GOGAT, glutamate synthase; TPC, total phenolic content; Na, sodium and K, potassium.

The correlation analysis results displayed a significant positive relation between nitrogen related enzymes (NR, nitrate reductase; GS, glutamine synthetase; and GOGAT, glutamate synthase), chlorophyll content (chla, chlb), and biomass accumulation (fresh and dry biomass). It showed that the increasing activities of nitrogen metabolism-related enzymes prompted nitrogen uptake and result in maximum biomass production. On the other hand, antioxidant enzymes activities (POD, peroxidase; CAT, catalase; APX, ascorbate peroxidase; SOD, superoxide dismutase; PPO, polyphenol oxidases; GTR, glutathione reductase) and protein, proline, and total phenolic content (TPC) had s strong negative correlation with nitrogen related enzymes (**Figure 8**).

## 4 Discussion

### 4.1 Exogenous GABA improved growth features of mungbean plants under saline stress

Increasing soil salinity is a major global issue in sustainable agriculture as it disrupts cellular and physiological processes at all growth stages (Arif et al., 2020). Salinity-induced reduction in plant growth may be due to low uptake of mineral nutrients (Askari-Khorasgani et al., 2017), such as potassium (K^+^), as a result of excess Na^+^ accumulation (Chakraborty et al., 2016). In the present study, a similar phenomenon was observed, as increased saline stress (SS) led to impaired growth features (stem height, root length, leaf area, stem-leaf-root fresh and dry weight; **Figures 1A–H**) and chlorophyll pigments (Chl a, Chl b; **Figures 2A, B**) of mungbean plants, suggesting hindrance of cell division. The decline in growth and biomass is correlated negatively with increased Na^+^ levels and excessive H_2_O_2_ production (**Figure 8**). Excessive H_2_O_2_ disrupts the plasma membrane causing ionic imbalance, inhibiting metabolic processes, eventually declining plant growth (Mittler et al., 2011; Kaya et al., 2019; Ullah et al., 2022b; Ullah et al., 2022c). In the present study, gamma-aminobutyric acid (GABA) was applied under SS to investigate whether or not it can mitigate salinity-induced adverse effects on mungbean growth and metabolism. We found that exogenous GABA relieved salt-induced reductions in plant growth features (**Figures 1A–H**). So, GABA might be an active growth regulator involved in the salinity-tolerance of mungbean plants, as has been reported in previous studies (Jin et al, 2018; Wang et al., 2015; Kalhora et al., 2018; Wu et al., 2020), which corroborated our findings. Hence, we suggest that exogenous GABA could increase the salinity-tolerance of mungbean by improving their growth under SS.

### 4.2 Exogenous GABA improved photosynthetic pigments under saline stress

The reduction in salinity-induced inhibitions of the photosynthetic pigments primarily inhibits plant growth. During chlorophyll biosynthesis in plants, a series of reactions take place, and when any of these reactions are disrupted, chlorophyll biosynthesis is affected (Min et al., 2006; Ali et al., 2022). In our study, increased SS significantly inhibited chlorophyll (Chl a and Chl b) concentration, which could be attributed to a specific symptom of SS-induced oxidative damage (Taïbi et al., 2016) and chlorophyllase inhibition/degradation (Chakhchar et al., 2018). An overabundance of H_2_O_2_ in mungbean plants under SS may also have contributed to the decrease in chlorophyll concentrations (Kaya et al., 2018). However, exogenous GABA improved chlorophyll concentration in SS-stressed plants, suggesting that GABA can mitigate the damaging effects of SS on photosynthetic pigments by reducing the production of H_2_O_2_ and improving plant growth. Similarly, GABA enhanced chlorophyll synthesis in maize plants under SS (Aljuaid and Ashour, 2022). Moreover, carotenoid concentrations significantly increased under 50 mM SS. Carotenoid is an auxiliary pigment that can dissipate excess light energy, making it an adaptive trait for dealing with salt stress (Duan et al., 2012). Exogenous GABA under 0 mM and 50 mM SS resulted in a significant increase in carotenoids compared to GABA-untreated counterparts (**Figure 2D**). Exogenous γ-aminobutyric acid (GABA) has been demonstrated to alleviate salinity-induced inhibition of plant growth by reducing chlorophyll degradation and enhancing photosynthetic capacity. For instance, in lettuce plants treated with exogenous GABA, salinity was less toxic to fresh and dry shoot masses than those untreated with GABA (Luo et al., 2011; Kalhora et al., 2018). So, GABA might be an active growth regulator involved in the salinity tolerance of mungbean plants by promoting their photosynthetic pigments and growth under SS.

### 4.3 Exogenous GABA alleviated Na^+^ toxicity by regulating Na^+^ concentration under saline stress

The excessive accumulation of Na^+^ caused by salinity stress leads to a decline in the uptake of essential nutrients like K^+^ from roots to leaves (Zhang et al., 2020). Increasing levels of Na^+^ and decreasing K^+^ in photosynthetic leaves may degrade chlorophyll and disrupt thylakoids (Bose et al., 2017). Plant salinity tolerance is characterized by its ability to lessen the accumulation of toxic Na^+^ ions in sensitive shoots (Niu et al., 2018). Hence, Na^+^ accumulation in tissues is often considered an indicator of the severity of salinity stress damage. In our study, increased salinity stress resulted in a significant increase in Na^+^ and a decline in K^+^ concentration in the leaves. Similar findings were reported for maize (Kaya and Ashraf, 2020; Aljuaid and Ashour, 2022), barley (Akhter et al., 2021), canola (Naveed et al., 2020), and soybean (Ullah et al., 2019). Further, salinity-induced decline in K^+^ ion resulted in low K^+^/Na^+^ ratio (**Figures 3B, C**). High salinity causes high pH in the rhizosphere, which reduces the availability of ions of nutrient elements, including K (Yang et al., 2007). Further, low K^+^ levels may also be caused by stress-induced repression of K^+^ absorption. Consequently, the low K^+^ and K^+^/Na^+^ ratio may also be attributed to K^+^ and Na^+^ competing for binding sites for cellular functions (Azooz et al., 2015; Ullah et al., 2019). In our study, the declined mungbean growth could be attributed to cellular membrane damage caused by osmotic, ionic, and pH-induced damage associated with a rise in Na^+^ ions and a decrease in beneficial K^+^ ions as a result of saline stress (Zhu, 2001; Chartzoulakis, 2005; Ullah et al., 2019; Aljuaid and Ashour, 2022; Noor et al., 2022). Several studies have been conducted on the effect of exogenous GABA on Na^+^ concentrations in plants, but whether GABA can directly reduce Na^+^ accumulation is unclear. Nevertheless, it has been demonstrated that *Arabidopsis* mutants with high GABA levels in their roots have significantly lower Na^+^ than mutants with low GABA levels (Su et al., 2019). In the present study, exogenous GABA treatments reduced Na^+^ levels in the leaves of mungbean plants, compared to their untreated GABA peers (**Figure 3A**). A study by Wu et al., 2020 found that exogenous GABA inhibits the absorption of Na^+^ ions by roots and reduced their transport to leaves. Further, GABA has been shown to improve plant salt tolerance by influencing ion membrane potential differences and osmoregulation, thereby enhancing ion transport (Seifikalhor et al., 2019). We, therefore, believe that exogenous GABA alleviates saline stress damage in mungbean plants by reducing Na^+^ in the leaves (**Figure 3A**). Numerous studies have demonstrated that GABA accumulation reduces Na^+^ uptake and reactive oxygen species (ROS) concentration, activates H^+^ ATPase, and inhibits K^+^ loss under stress conditions (Shi et al., 2010; Li et al., 2019, 2020; Su et al., 2019; Wu et al., 2021; Aljuaid and Ashour, 2022). Therefore, we suggest that GABA-induced reduction in Na^+^ concentration was associated with increased K^+^ levels and a high Na^+^/K^+^ ratio, which is beneficial for salinity adaptation.

### 4.4 Exogenous GABA improved salinity tolerance by regulating the antioxidant potential of mungbean plants

The osmotic and oxidative stresses caused by salinity impair plant metabolism (Ma et al., 2018), leading to the accumulation of lipid peroxides and membrane damage (Mittler et al., 2004). Compared to controls, we observed significantly higher levels of MDA and H_2_O_2_ during all levels of saline stress. Similar results were reported for chufa (Ullah et al., 2022b), maize (Aljuaid and Ashour, 2022), barley (Akhter et al., 2021), canola (Naveed et al., 2020), and pepper (Kaya et al., 2020b). It is well known that plants possess a highly specialized antioxidant defense system that is capable of scavenging reactive oxygen species (ROS) under stress conditions, including salinity. in the present study, saline-treated mungbean plants displayed significantly greater levels of antioxidant enzymes, compared to control mungbean plants. For example, compared to the control, the activities of SOD, POD, APX, PPO, and GTR, increased with increasing saline stress levels, indicating that it is a solid antioxidant defense mechanism for detoxifying the excessive ROS (Munns and Tester, 2008; Polash et al., 2019). Several studies have reported the upregulation of SOD, POD, APX, PPO, GTR, and CAT in plants under salinity stress (Luo et al., 2011; Weisany et al., 2012; Farhangi-Abriz and Torabian, 2017; Aljuaid and Ashour, 2022), which corroborate our findings. It is suggested that salinity-treated mungbean plants upregulate their antioxidant enzymes to scavenge ROS at the expense of growth and biomass reduction as more energy has been devoted to antioxidant mechanisms instead of organ development. Further, we found that exogenous GABA significantly reduced H_2_O_2_ and MDA under both saline stress levels, where its effect on antioxidant enzymes was only significant under 50 mM saline stress. It has been demonstrated that GABA exhibits ROS scavenging potentials and can contribute to stress mitigation (Liu et al., 2011; Aljuaid and Ashour, 2022). For instance, GABA inhibits MDA, an indicator of lipid oxidation (Deng et al., 2010; Aljuaid and Ashour, 2022). However, it is unclear whether GABA can directly scavenge ROS to alleviate stress. Still, several studies reported that exogenous GABA plays a key role in scavenging ROS and modulating the activity of antioxidant enzymes (Renault et al., 2010; Barbosa et al., 2010; Aljuaid and Ashour, 2022). We suggest that exogenous GABA protected mungbean plants from salinity-associated oxidative stress damage and enhance their tolerance by reducing lipid peroxidation and upregulating enzymatic antioxidant mechanisms.

### 4.5 Exogenous GABA improved nitrogen concentration by regulating N-metabolizing enzymes in mungbean plants

Regulation of nitrogen (N) metabolism is essential for salt tolerance, and salinity and N nutrition interact intricately (Läuchli and Lüttge, 2002). In this study, mungbean plants subjected to saline stress had lower N content and, along with declined activities of nitrate reductase (NR), glutamine synthetase (GS), and glutamine oxoglutarate aminotransferase/glutamate synthetase (GOGAT) (**Figure 6A**-D). The salinity-induced decline in nitrogen assimilation has been reported for tomato (Debouba et al., 2006), Chinese cottonwood (Meng et al., 2016), soybean (Ullah et al., 2019), and chufa (Ullah et al., 2022b), which corroborate our findings. We suggest that salt ions impaired nitrogen metabolism in mungbean plants by inhibiting NR activity which prevents GS/GOGAT from supplying NH_4_^+^ to the amino acid synthesis pathway (Debouba et al., 2006; Meng et al., 2016). The decrease in N assimilation and the synthesis of amino acids and proteins can reduce plants’ dry weight (Queiroz et al., 2012). However, exogenous GABA treatments improved nitrogen concentration and activities of NR, GS and GOGAT under salt and controlled condition, compared to their untreated peers. However, the effect was only significant under 50 mM saline stress. GABA has been shown to play a role in buffering N metabolism (Salah et al., 2019). For example, GABA may influence plants’ growth and stress responses by regulating their carbon and nitrogen metabolism (Chen et al., 2020). A recent study found that plants treated with GABA displayed increased nitrogen metabolism, as illustrated by increased nitrogen concentration and its metabolizing enzymes (Khanna et al., 2021), which support our findings. Therefore, we suggest that exogenous GABA enhances salinity stress tolerance of mungbean plants by regulating the key enzymes of N assimilation and increase N concentrations, resulting in improved growth and biomass.

### 4.6 Exogenous GABA, regulated biochemical changes in mungbean plants

In the present study, significantly higher concentration of soluble sugar recorded at 50 mM, compared to control condition. Under stress condition, plants use soluble sugars to maintain osmotic and ionic homeostasis, limit chlorophyll destruction and water loss, remove excess ROS, stabilize proteins and membrane structures, regulate cell division, and control gene transcription (Sami et al., 2016). Moreover, there was a significant increase in proline, soluble protein and total phenolic concentration with increasing saline stress levels compared to the control condition. The accumulation of proline might have contributed to the protection of photosynthetic apparatus elimination of excessive ROS to stabilize membranes, enzymes, and proteins in salinity-treated mungbean plants (Verdoy et al., 2006; Ahmad et al., 2016). In other studies, proteins have also been shown to act as osmotins and contribute to salt stress tolerance (Zhang et al., 2013). Furthermore, plants have the ability to upregulate small molecules of protein that can be used for storing N and can be mobilized rapidly during a period of stress relief (Singh et al., 1987). Osmotic adjustment may also be mediated by these proteins (Ashra and Harris, 2004). In addition, phenolic compounds play an important role in protecting plants from both biotic and abiotic stress (Ma et al., 2018; 2019). The application of exogenous GABA further increased soluble sugar, proline, soluble protein, and total phenolic content, suggesting that the GABA protected the mungbean plants from salt stress and thus enhanced their salinity tolerance.

## 5 Conclusion

Exogenous GABA was used to test whether it could mitigate the adverse effects of saline stress on mungbean plants’ growth and physio-biochemical attributes. Increased saline stress adversely affected the growth, biomass, and physiological metabolism of our test species. However, exogenous GABA decreased the damage caused by salinity stress on mungbean plants by promoting growth and physiological metabolism. For example, exogenous GABA causes (i) reduced Na^+^ concentration, (ii) increased K^+^, which resulted in a higher K^+^/Na^+^ ratio, (iii) promoted photosynthetic pigment biosynthesis, (iv) reduced H_2_O_2_ and MDA concentration by upregulating enzymatic antioxidant potential (v) improved N concentration and activities of N-metabolizing enzymes and (vi) enhanced osmolytes accumulations. Our findings suggest that GABA can mitigate the salinity-associated morpho-physio-biochemical damages in mungbean plants. Therefore, applying GABA to mungbean plants could effectively reduce salinity-induced adverse effects on the growth and yield of mungbean plants in salinity-affected areas worldwide. Future research should investigate whether exogenous GABA can positively influence the nutrient composition of mungbean at deeper molecular levels to offer solutions to its low productivity in salt-affected areas.

## Data availability statement

The raw data supporting the conclusions of this article will be made available by the authors, without undue reservation.

## Author contributions

Conceptualization: AU, SU, Data curation: AU. Formal analysis: AU, MA, IA, SB, SE and JN. Investigation: AU, JN. Methodology: AU and SU. Project administration: SU. Resources: AU and SU. Software: AU, MA, KS, JN, and HJ. Supervision: SU. Validation: AU, SU, and Visualization: JN, KS, KS, HJ, and MA. Writing - original draft: AU, JN, IA, SB, SE and HA: Writing - review and editing. All authors contributed to the article and approved the submitted version.
