# Supplementary material for: Exogenous γ-aminobutyric acid (GABA) mitigated salinity-induced impairments in mungbean plants by regulating their nitrogen metabolism and antioxidant potential
Source: Front Plant Sci. 2023 Jan 18;13:1081188. doi: 10.3389/fpls.2022.1081188 (PMC9897288; doi:10.3389/fpls.2022.1081188)
Supplement: Supplementary file 1 [file Table_1.docx]

GABA concentrations ranging from 0.25 mM to 1.5 mM were tested in a preliminary growth experiment on 5-days old mungbean seedlings. The optimal concentration of GABA was determined based on the improved growth of seedlings in a solution containing 50 mM of saline stress.

Effect of different GABA concentrations (0.35-1.5 mM) on 5-days old mungbean seedlings under saline stress (96 h)

| Treatments | Plumule length (cm) | Radicle length (cm) | Plumule fresh weight (g) | Plumule dry weight (g) | Radicle fresh weight (g) | Radicle dry weight (g) |
| --- | --- | --- | --- | --- | --- | --- |
| 0 mM saline stress | 6.733 ± 0.097a | 8.767 ± 0.208a | 0.369 ± 0.003a | 0.176 ± 0.008a | 0.063 ± 0.002a | 0.027 ± 0.001a |
| 50 mM saline stress | 4.870 ± 0.076d | 6.233 ± 0.153d | 0.203 ± 0.002e | 0.121 ± 0.019c | 0.047 ± 0.002d | 0.019 ± 0.001c |
| 50 mM saline stress + 0.25 mM GABA | 4.843 ± 0.267d | 6.600 ± 0.100c | 0.234 ± 0.003d | 0.124 ± 0.011c | 0.049 ± 0.002d | 0.019 ± 0.001c |
| 50 mM saline stress + 0.5 mM GABA | 5.053 ± 0.270d | 6.833 ± 0.208c | 0.236 ± 0.003d | 0.131 ± 0.005c | 0.052 ± 0.001c | 0.020 ± 0.000c |
| 50 mM saline stress + 1 mM GABA | 5.800 ± 0.151c | 6.900 ± 0.100c | 0.268 ± 0.002c | 0.139 ± 0.004bc | 0.055 ± 0.001c | 0.023 ± 0.000b |
| 50 mM saline stress +1.5 mM GABA | 6.183 ± 0.085b | 7.867 ± 0.208b | 0.337 ± 0.004b | 0.152 ± 0.013b | 0.059 ± 0.001b | 0.025 ± 0.001b |

Values are mean ± SE. Different letters indicate significant differences at *P* < 0.05 based on Duncan’s multiple range test.
